# Reconstruction of delayed scleral flap melting with bovine pericardium after trabeculectomy with mitomycin C

**DOI:** 10.3205/oc000066

**Published:** 2017-06-27

**Authors:** Inês Coutinho, Diana Silva, Mafalda Mota, Maria Lisboa, Fernando Trancoso Vaz, Isabel Prieto

**Affiliations:** 1 Ophthalmology Department, Hospital Prof. Doutor Fernando Fonseca, Amadora, Portugal

**Keywords:** trabeculectomy, glaucoma, hypotony maculopathy, scleral flap

## Abstract

**Aim:** To present a challenging case of hypotony after trabeculectomy and its treatment.

**Case description:** A 22-year-old woman with juvenile glaucoma underwent a conventional trabeculectomy with mitomycin C on the right eye (OD).

In the immediate postoperative period, we observed a hyperfiltration bleb with hypotony refractory to conservative measures leading to hypotony maculopathy.

A surgical revision with scleral flap resuture and conjunctival graft was performed with a satisfactory result and resolution of hypotony maculopathy.

After two years, the patient complained of low visual acuity (VA) of the OD. During examination, we observed a fine and avascular bleb with Seidel and visualization of the underlying uveal tissue, an intraocular pressure (IOP) of 5 mmHg, and chorioretinal folds.

A new revision of the trabeculectomy was performed. During the procedure, it was not possible to identify the scleral flap, so the fistula was closed with a patch of collagenous membrane derived from bovine pericardium (Tutopatch^®^ graft).

A good clinical evolution occurred. After 2 months, IOP was 15 mmHg without Seidel or changes in the fundus and VA was 20/20. After 8 months of follow-up, the IOP remains stable without further complaints.

**Conclusion:** This case illustrates the difficulties faced in the management of a common complication of trabeculectomy and highlights some of the options available for its treatment. There are few reports of scleral melting after trabeculectomy. However, trauma and scleral necrosis associated with mitomycin are listed as the main causes.

The use of a scleral patch derived from bovine pericardium allows effective suturing and closure of the aqueous leak.

## Introduction

Although associated to some possible complications, trabeculectomy remains the mainstay of surgical management of glaucoma [1], [2]. The advent of antimetabolite adjunctive therapy has increased the success rate of this procedure due to his antifibrotic action. However, this gain comes with some complications associated.

The more frequent complication of mitomycin C is postoperative hypotony, which may be defined as an intraocular pressure (IOP) less than 6 mmHg or the lowest IOP that leads to pathologic functional and structural changes [3].

Early hypotony (first two weeks after surgery) occurs most of the time secondary to bleb leaks resulting from poor wound closure or from overfiltration. In late hypotony, the main causes are ischemic, avascular, and thin bleb or an overfiltrating bleb. The management of late hypotony is difficult and the prognosis for visual recovery depends on its duration [4].

Maceration and melting of the scleral bed can also be other possible causes of hypotony, but much less frequent [2], [4].

Hypotony maculopathy is a serious complication that is characterized by a decrease in visual acuity caused by macular folds, retinal edema, papilledema, and vascular tortuosity. If left untreated, it may cause photoreceptor damage, which can limit the recovery of the visual function even after a normal IOP has been restored [3].

**Figure 1:**
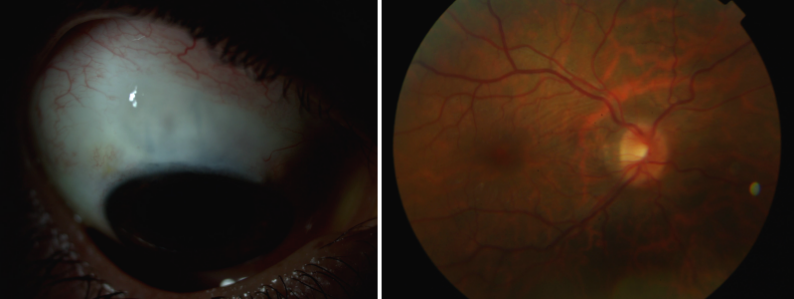
Thin filtration bleb and chorioretinal folds

**Figure 2:**
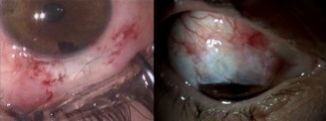
Suture of the scleral flap (left) and conjunctival graft (right)

Treatment options of leaking and overflitrating blebs include non-surgical and surgical therapies [2]. The non-surgical treatment includes aqueous suppressants, corticosteroids reduction, pressure patching or bandage contact lens, cyanoacrylate glue, laser application on bleb, and injection of autologous blood beneath the bleb. The surgical bleb revision can be performed by conjunctival advancement with or without bleb excision, scleral flap re-suture, free conjunctival flap or patch, amniotic membrane graft, and donor scleral or corneal patching [2], [5].(Fig. 1)(Fig. 2)

This case report aims to describe one case of scleral melting and hypotony maculopathy after a trabeculectomy with mitomycin C as well as to report the resolution chosen and the evolution of the patient. 

## Case description

A 22-year-old Caucasian woman with juvenile glaucoma underwent a conventional trabeculectomy with mitomycin C 0.4 mg/mL OD. The mitomycin was applied beneath conjunctiva/Tenon’s capsule and scleral flap for 3 minutes after performing a 4x4 mm limbal-based scleral flap, and prior to sclerotomy. The surgery took place without any complications. 

In the immediate postoperative period, we observed a hyperfiltrating bleb without Seidel phenomenon. This led to hypotony refractory to conservative measures such as pressure patching, decreased of corticosteroids dosage and use of aqueous suppressants and three weeks after, she developed hypotony maculopathy with low visual acuity (VA = 20/60 OD) (Figure 1 (Fig. 1)).

A surgical revision with 2 additional sutures in the scleral flap was performed. Three weeks later, the hypotony remained as well as the thin conjunctiva, so we decided for a conjunctival autograft from the inferior bulbar conjunctiva. The result was satisfactory and the hypotony resolved (Figure 2 (Fig. 2)), although, in the early postoperative period, there was a history of minor blunt ocular trauma without further complications.

**Figure 3:**
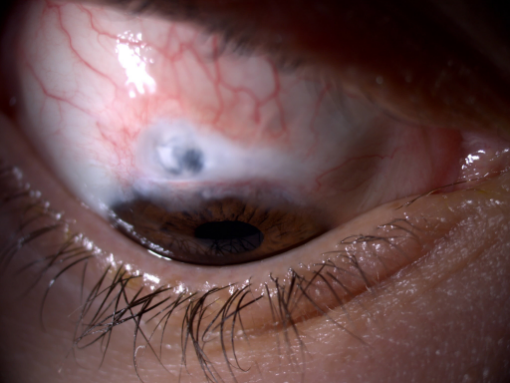
Avascular bleb and visualization of the underlying uveal tissue

After two years, the patient attended our appointment due to low visual acuity. On examination, VA OD was 20/200, IOP was 5mm Hg with a thin, avascular and transudative bleb, visualizing the underlying uveal tissue with chorioretinal folds (Figure 3 (Fig. 3)). Connective tissue diseases were excluded. 

**Figure 4:**
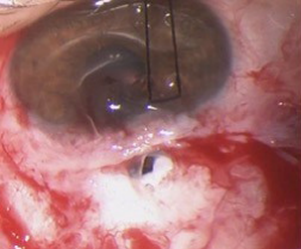
Absence of scleral flap

**Figure 5:**
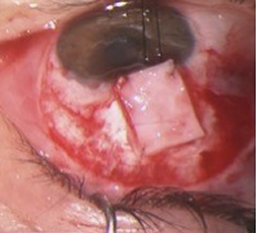
Tutopatch graft^®^

A new trabeculectomy revision was performed. During the procedure, it was not possible to identify the scleral flap (Figure 4 (Fig. 4)), so we used 2 layers of Tutopatch^®^ (a collagenous membrane derived from solvent preserved irradiated bovine pericardium) to close the fistula (Figure 5 (Fig. 5)). 

**Figure 6:**
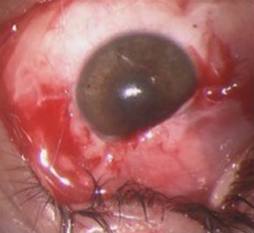
Conjunctiva over the graft

The 2 layers of Tutopatch^®^ were attached to each other with fibrin sealant and the graft sutured to the adjacent sclera with 10-0 nylon and covered by the conjunctiva (Figure 6 (Fig. 6)). 

**Figure 7:**
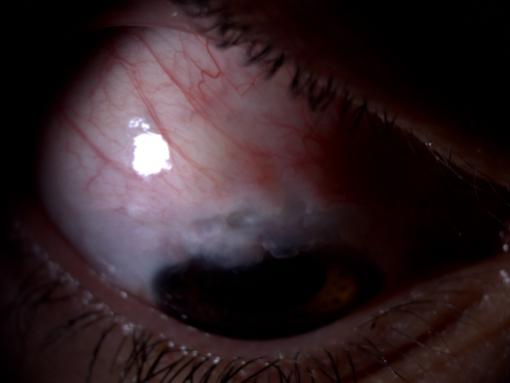
After 8 months

A good clinical evolution occurred and after 2 months, IOP was 15 mmHg without Seidel phenomenon or changes in the fundus and VA was 20/20 OD (Figure 7 (Fig. 7)). After 8 months of follow-up, the clinical situation remains stable.

## Discussion/conclusion

Hypotony is a common complication after trabeculectomy and its resolution is not always easy [5], [6].

Since 2008, in our department, we are performing Moorfields Safer Surgery trabeculectomy developed by Professor Peng Khaw from Moorfields Eye Hospital. The objectives of this technique are: to increase intra- and postoperative safety (stability of the anterior chamber during surgery, control early drainage flow and reduce hypotony), increase reproducibility of surgery (standard sclerotomy, tightening of adjustable sutures under continuous infusion), and decrease long-term bleb failure (fornix based approach, broad conjunctival dissection and diffuse antifibrotic application if indicated).

In the current literature, there are few reports of scleral melting after trabeculectomy, but the main causes associated are trauma and mitomycin use and may occur early or even years after surgery [7], [8]. Inflammation, ischemia, and apoptosis are the main triggers for scleral thinning and necrosis. In this case, several factors may have contributed to the melting of the scleral flap, such as: local trauma induced by multiple surgeries, use of cautery and mitomycin C on scleral bed as well as use of topical corticoids in the postoperative period, which have a known effect in reducing collagen synthesis [9], [10].

A patch of collagenous membrane derived from bovine pericardium above the fistula seems an adequate therapy to treat a scleral flap defect and a secondary over-filtering bleb [11].

This case can be relevant to highlight this rare complication after trabeculectomy as well as its management. 

## Notes

### Competing interests

The authors declare that they have no competing interests.
